# Impact of Stationary Brush Quantity on Brush Use in Group-Housed Dairy Heifers

**DOI:** 10.3390/ani12080972

**Published:** 2022-04-08

**Authors:** Faith S. Reyes, Amanda R. Gimenez, Kaylee M. Anderson, Emily K. Miller-Cushon, Joao R. Dorea, Jennifer M. C. Van Os

**Affiliations:** 1 Department of Animal and Dairy Sciences, University of Wisconsin-Madison, Madison, WI 53706, USA; fbaier@wisc.edu (F.S.R.); joao.dorea@wisc.edu (J.R.D.); 2 School of Veterinary Medicine, University of Wisconsin-Madison, Madison, WI 53706, USA; agimenez@wisc.edu (A.R.G.); kcoel@wisc.edu (K.M.A.); 3 Department of Animal Sciences, University of Florida, Gainesville, FL 32611, USA; emillerc@ufl.edu

**Keywords:** grooming, oral behavior, competition, bout criteria

## Abstract

**Simple Summary:**

Grooming and oral manipulation are motivated natural behaviors for cattle and can be practically facilitated by provision of stationary brushes. It is unknown how many brushes should be provided to a group to allow for ample opportunity for use. Our main objectives were to evaluate the effect of the number of brushes on use and competition in group-housed, weaned dairy heifers naïve to brushes. Brush quantity did not impact overall durations of brush use and competition, but the provision of four vs. two brushes per group of eight heifers resulted in longer bouts, suggesting better opportunity for uninterrupted use. The continued use of brushes over time by all heifers supports the importance of providing appropriate outlets for natural behaviors, such as grooming and oral manipulation, to promote animal welfare.

**Abstract:**

Our objectives were to evaluate the effect of stationary brush quantity on brush use and competition in weaned dairy heifers naïve to brushes. Sixty-three Holstein heifers (95 ± 5.7 days old) were housed in groups of eight (with the exception of 1 group of 7) with two or four stationary brushes (*n* = 4 groups/treatment). Brush-directed behaviors of grooming, oral manipulation, and displacements were recorded continuously for all heifers 0–6, 18–24, 120–126 and 138–144 h after brush exposure. Linear mixed models were used to evaluate the effects of brush quantity and exposure duration. Total brush use and competition were not affected by brush quantity, but heifers with access to more brushes used them for longer bouts, suggesting greater opportunity for uninterrupted use. Total brush use was greater in the first and final 6 h observation periods, which was driven by the greatest duration of oral manipulation and grooming in those respective periods. The continued use of brushes by all heifers in the final period indicates the importance of providing appropriate outlets for these natural behaviors to promote animal welfare. The effect of brush quantity on bout characteristics suggests that brush use was less restricted with four compared to two brushes per eight heifers.

## 1. Introduction

Grooming is a motivated behavioral need for cattle that can be facilitated with objects in the environment, such as brushes [1,2]. Grooming can improve cleanliness [3,4], remove parasites [5], satisfy a natural behavior [6], and may reduce boredom, stress, and frustration [7]. The use of either mechanical rotating or stationary brushes has been documented in cattle of several age groups, including pre-weaned calves [8,9,10], weaned heifers [11,12,13], periparturient cows [14,15], dry cows [16], and lactating cows [2,17]. The provision of appropriate resources to perform this highly motivated behavior [1] may thus improve the welfare of cattle, especially weaned dairy heifers, who are often housed in relatively barren confined environments.

Stationary brushes can be a more economical option than mechanical rotating brushes [18]. Although rotating brushes are designed to allow cattle to groom harder to reach areas of the body (i.e., the back) [2,19], young cattle primarily choose to brush their head and neck compared to other body areas, regardless of brush type (vertically mounted rotating brushes [8,20]; stationary brushes [9,13]). Furthermore, stationary brushes also provide an outlet for oral manipulation [10,13], which has not been reported for mechanical brushes. In addition to grooming, this behavior is also important, especially for young cattle, for whom the provision of stationary brushes has been shown to decrease the abnormal oral behaviors of sucking on pen structures [10] and tongue rolling [21]. Despite the current evidence that the provision of brushes offers appropriate outlets for cattle to perform these important natural behaviors, our understanding of brush use based on the availability of brushes in a group setting is limited.

It is unknown how many brushes are needed to provide group-housed heifers with adequate opportunities for use. Competition for brush access (i.e., displacement events) has been reported for mechanical brushes [2,16,22], but rarely for stationary brushes [13]. The majority of previous studies provided only a single brush to groups of cattle (i.e., 4 pre-weaned dairy calves [9], 19 to 20 weaned dairy calves [12], 9 beef steers [21], 4 or 8 beef steers [23], and 20 lactating dairy cows [16]). Van Os et al. [13] provided four brushes to groups of four weaned heifers in an open bedded-pack pen, followed by four brushes per pair of heifers in a freestall pen, but no comparison of brush use was performed depending on the quantity of brushes offered. To our knowledge, no studies have directly evaluated the role of stocking density on brush use or competition. Information about how brush quantity affects resource competition could improve our understanding of brush use and inform management decisions related to providing brushes to cattle. 

In addition to group dynamics, temporal patterns of brush use also play a role in adequate access to brushes. Cattle exhibit varying temporal patterns when using brushes, both across days as well as relating to specific events or bouts. For example, weaned dairy heifers exhibited the greatest use of stationary brushes on the first day of observation, with use continuing throughout the 13 day observation period [13]. Similar patterns were observed in beef steers, with the greatest stationary brush use on the first day of observation (day 1 of exposure [24]; day 2 of exposure [21]) and continued use throughout the observation periods (ending on day 22 and day 64, respectively). Many studies have reported brush use as average durations during an observation period (e.g., across 24 h). In contrast, brush visit or bout characteristics describing the temporal patterns of brush use events have only been reported in pre-weaned calves [8,9], but not for other age classes of dairy cattle. Similarly to feeding behavior [25], brush use often occurs for short durations separated by short breaks. Calculating bout characteristics to group events into bouts can provide a more informative depiction of brush use duration and frequency (e.g., ref. [9]) than reporting raw observations (e.g., ref. [8]).

Our main objective was to evaluate the effect of the number of stationary brushes on brush use and competition in group-housed, weaned dairy heifers naïve to brushes. This included evaluating the time scale of brush use across hours of exposure, along with the bout characteristics of brush use within observation periods. We predicted that the provision of more brushes would result in less competition along with more frequent and longer bouts based on the greater opportunity for brush access, resulting in greater total brush use. We also predicted that individual heifers who were more successful at displacing other heifers from brushes would exhibit greater brush use. Finally, we predicted that brush use would decrease after the initial 6 h observation period when the brushes were most novel.

## 2. Materials and Methods

### 2.1. Animals, Housing, and Treatments

The study was conducted from June to August 2019 at the University of Wisconsin—Madison (UW-Madison) Marshfield Agricultural Research Station in Marshfield, WI. All procedures were approved by the Institutional Animal Care and Use Committee of University of Wisconsin—Madison (protocol A006133-A01, approved 10 May 2019).

Sixty-three Holstein heifers were housed in groups of 8, with the exception of one pen with 7 heifers (due to one heifer dying before arrival for reasons unrelated to the study). Each group was determined by birth order and previously housed together in outdoor group hutches before being moved to the treatment pens. Groups of heifers were moved to the treatment pens at approximately the same age. Therefore, heifer age remained relatively consistent among and within groups. Four experimental pens were filled sequentially by arrival date (95 ± 5.7 days old upon arrival, mean ± SD; 118.3 ± 11.9 kg bodyweight, excluding the pen of 7 heifers, for which bodyweight data were unavailable), with each pen used for 2 groups.

Each pen included a 4.9 m × 4.9 m open lying area bedded with straw (0.2 m bedding height; added on an as-needed basis). Two opposite sides of each pen had 1.6 m-tall wooden walls and a feed bunk containing 9 self-locking headlocks, opposite a metal gate. Twice daily, heifers were fed grain (0730 and 1515 h) and unchopped grass hay (0830 and 1600 h). All heifers, except those in the pen of 7, were also enrolled in a separate, larger nutrition trial involving diets that varied in formulation of feed additives [26]. We do not expect this factor affected brush use. Water was provided ad libitum from one self-filling water trough per pen. 

**Figure 1:**
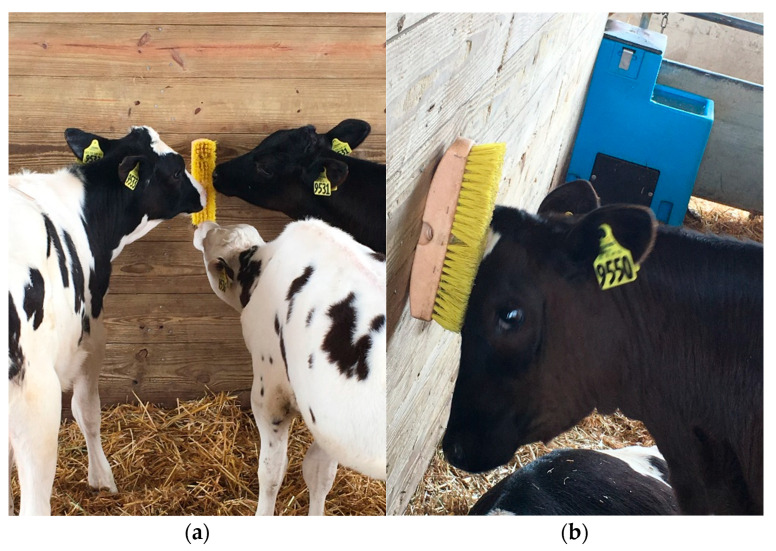
Group-housed heifers were provided with either two or four wash brushes per pen, mounted on two opposite wooden plank walls of the pen. Brush-directed behavior was observed for all heifers via continuous video recordings and coded as (**a**) oral manipulation, defined as contact between the mouth or tongue and the brush and (**b**) grooming, defined as rubbing the head, neck, or body against the brush.

**Figure 2:**
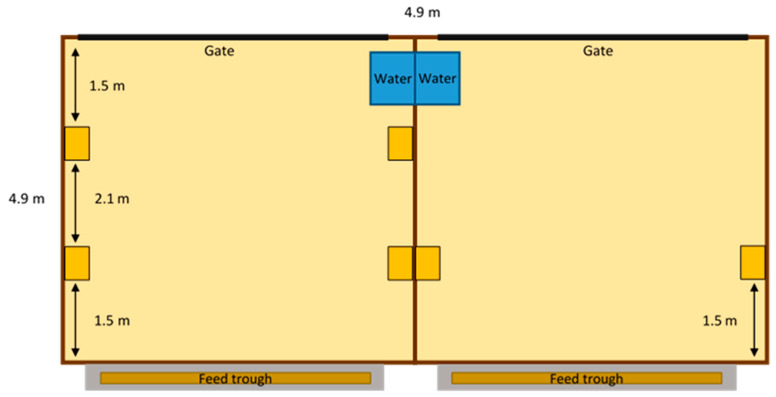
Pen diagram for each of the two brush treatments, providing two or four brushes to group-housed, naïve heifers. The yellow boxes represent an individual brush. Solid wooden walls separated the 4 pens in the barn. Note: This diagram is not drawn to scale.

The experimental treatment was the number of brushes provided in each pen (2 vs. 4/pen; Pro Series Wash Brush, 25.4 cm-long × 6.0 cm-wide with 3.8 cm-long bristles, described as “stiff” by the manufacturer, Camco Manufacturing; Figure 1), with brush treatment alternating between adjacent pens (Figure 2). A total of *n* = 4 groups/treatment were tested; the 2-brush treatment had 3 groups of 8 heifers and one group of 7 heifers, and the 4-brush treatment had 4 groups of 8 heifers. Treatments were assigned alternately to groups of heifers by filling the next sequential unoccupied pen. Brushes were mounted vertically on the 2 wooden walls in each pen at a height of 0.9 m, measured from the floor beneath the bedding to the bottom of the brush. In the 2-brush pens, the brushes were mounted 1.5 m from the feed bunk, based on previous work showing that dairy cattle use brushes located near the feed bunk [13,27]; in the 4-brush pens, the additional 2 brushes were mounted 1.5 m from the gate.

### 2.2. Power Analysis

Sample size justification was performed using data from a previous study [13], which examined brush use in group-housed heifers (*n* = 13 groups of 4 heifers each). The durations of brush use, reported as oral manipulation (mean range of 7.5 to 14.8 min, SD = 0.5 to 5.1), grooming (mean range of 11 to 27.2 min, SD = 3.4 to 11.4), and total brush use (mean range of 21.0 to 45.9 min, SD = 2.8 to 17.6) translated to very large effect sizes (Cohen’s *d* = 1.4 to 1.5), which resulted in requiring a sample size of *n* = 7 to 8 groups of heifers/treatment. However, the present aim was to examine behavior within larger groups (7 to 8 heifers/group), whereas behavioral observation for the previous study was based on 2 focal heifers within each group. As described by St-Pierre [28], the number of replicates needed to achieve a given power is decreased when larger groups are the experimental unit, as variance among pens is generally less than the variance of animals within pens. In consideration of decreased variability when using larger groups, we enrolled 4 groups of heifers per treatment.

### 2.3. Measures

To characterize heifer behavior, video was recorded using 4 video cameras (3 MP ProHDDome IP Fixed Outdoor Camera, Amcrest, with internal SD cards) mounted 3.5 m high on the barn wall closest to the gates. Recorded files were off-loaded from the cameras to external hard drives for observation. Behavior was coded for each individual heifer from video for four 6 h periods on the first and sixth days relative to entering the pen on day 1, defined as follows, with approximate times of day: period 1: 0–6 h after exposure (day 1, 1230–1830 h); period 2: 18–24 h after exposure (day 1, 0630–1230 h); period 3: 120–126 h after exposure (day 6, 1230–1830 h); period 4: 138–144 h after exposure (day 6, 0630–1230 h). For one group assigned to a 4-brush pen, video data on day 6 (120–126 and 138–144 h of brush exposure) were lost due to hard drive malfunction. Behavior was only observed during daylight hours to ensure accuracy of observations. Two trained observers watched the recordings with a video-supported player (Amcrest Smart Player, 1080 HD-CVI) and coded behaviors in Excel spreadsheets for all heifers continuously during those periods. Individual heifers were identified by their coat patterns. Specific brushes were identified by the location in each pen. Brush-directed behavior was recorded as two different types: (1) oral manipulation—contact between the mouth or tongue and a brush (Figure 1A)—and (2) grooming—rubbing the head, neck, or body against a brush (Figure 1B). The duration of each behavior was determined by recording the start and stop times to the nearest second. Displacement events occurring at each brush were also recorded when one heifer’s contact with the brush (receiver) ended due to physical contact from another heifer (actor). Inter-observer reliability for all brush-directed behaviors was determined by comparing a subset of data between the two observers using linear regression (PROC REG; SAS software version 9.4; SAS Institute, Cary, NC, USA). The coefficient of determination (R^2^) was ≥0.89, and the slope and intercept did not differ from 1 and 0, respectively (*p* > 0.05), which indicated good agreement and lack of systematic bias.

### 2.4. Statistical Analysis

Latency to initially contact any brush was calculated on an individual heifer basis relative to their respective entry time into the pen. Latency-related parameters are reported only descriptively. Total brush use was calculated as the sum of brush grooming and oral manipulation. A competitive index was calculated for each heifer using her frequency of initiated displacements (actor role) divided by the total number of displacements she was involved in (actor and receiver roles), multiplied by 100; one heifer (in the 4-brush treatment) uninvolved in displacements was excluded. 

**Figure 3:**
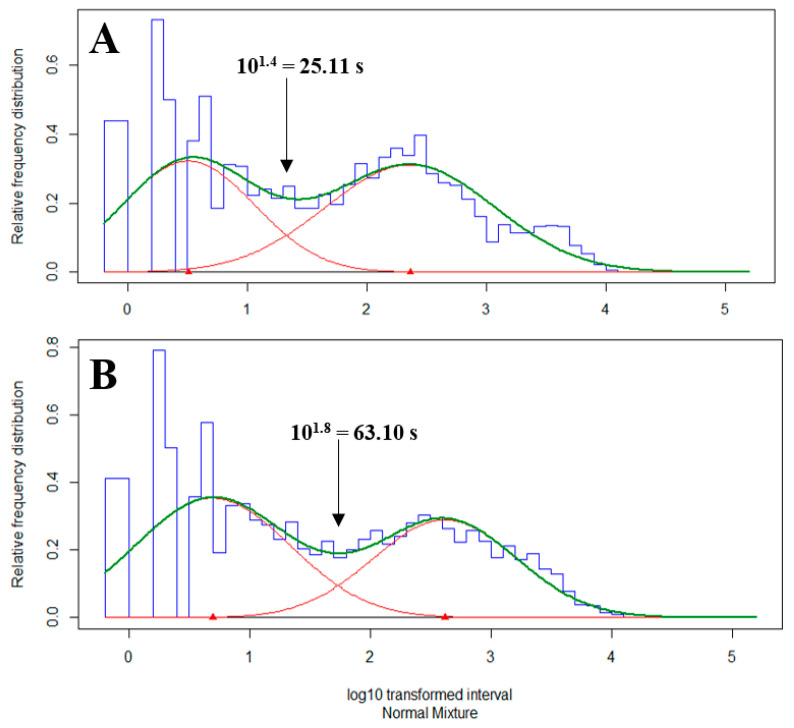
Log_10_-transformed relative frequency distributions of the intervals between the events of heifers using stationary brushes, fitted with mixture normal distributions. Data presented are summarized for the (**A**) novel period (0–6 and 18–24 h of exposure, pooled across 63 heifers) and (**B**) non-novel period (120–126 and 138–144 h of exposure, pooled across 55 heifers). The blue bars represent the frequency of each log_10_-transformed inter-bout interval. The red lines represent the contribution of individual distributions to the overall probability density (green line).

To evaluate the bout characteristics of brush use, bout analysis was performed for total brush use in each of the four 6 h observation periods, pooled among all individual heifers. The analysis was performed as described by Horvath and Miller-Cushon [9] and DeVries et al. [25]. In brief, interval durations between each brush use event for each heifer were summarized and converted to log_10_-transformed frequency distributions to calculate the inter-bout criteria. The inter-bout criteria were calculated by fitting a mixture of two normal distributions to the log_10_ distributions of brush-use intervals using exact maximum likelihood to determine the point at which the distribution curve of within-bout (intra-bout) intervals intersected the distribution curve of between-bout (inter-bout) intervals (R package mixdist; in [29]). The calculated inter-bout criterion defines the interval between brush use bouts, accounting for behavioral bouts comprising multiple single events separated by short breaks. This criterion establishes a threshold such that brush use events separated by pauses shorter than the criterion are considered within the same bout, whereas those events separated by breaks longer than the criterion are considered as occurring in separate bouts. An individual inter-bout criterion was calculated for each 6 h period and pooled among all individual heifers. Similar inter-bout criteria were calculated for periods 1 (19.95 s) and 2 (39.81 s), when the brushes were relatively novel to the heifers, compared to periods 3 (125.95 s) and 4 (100 s). Therefore, two criteria were calculated, defined as follows: novel period: 0–6 and 18–24 h of exposure (periods 1 and 2), calculated as 25.11 s (Figure 3A); non-novel period: 120–126 and 138–144 h of exposure (periods 3 and 4), calculated as 63.10 s (Figure 3B). Using the respective bout criteria for the novel and non-novel observation periods, brush use bout characteristics were calculated for each heifer and summarized at the group level for each of the four observation periods. Two heifers (one each in the 2- and 4-brush treatments) in the novel period and 2 heifers (both assigned to the 2-brush treatment, but in different groups) in the non-novel period were excluded from the calculations of total time and bout duration due to either only 1 event recorded within that period or none of the events involving intra-bout intervals (i.e., intervals less than the inter-bout criterion). Bout characteristics were defined as total time (sum of duration of observed brush use and intra-bout intervals shorter than the inter-bout criterion), bout frequency (number of intervals between bouts, as defined by the inter-bout criterion), and bout duration (total time divided by bout frequency). Total time was used only for calculation of bout frequency and is not reported in the results. 

All statistical analysis was performed using R software (v. 3.6.1, RStudio, Boston, MA, USA). Linear mixed models were used to evaluate the effects of brush quantity and time of exposure on brush use (grooming, oral manipulation, and total brush use, along with the bout characteristics for total brush use) and competitive displacement events. These models included fixed effects of treatment (2 vs. 4 brushes/pen), period (0–6, 18–24, 120–126, or 138–144 h of brush exposure), and the interaction between treatment and period, and a random effect of group to account for the repeated measures of period. A Kenward–Roger adjustment was performed for all analyses of variance of linear mixed models. The competitive index had only one value per group (no effects of period), and thus the linear model for this variable included the fixed effect of only treatment. Residuals were assessed for normality and equal variance visually using graphs and numerically using the Shapiro–Wilk test for normality. The experimental unit was defined as a group of heifers (*n* = 4 groups/treatment) for all variables. For the competitive index, Pearson correlations were performed at the individual heifer level between the duration of total brush use (averaged across the four 6 h periods) and competitive index values; these relationships were evaluated both pooled between treatments and separately by treatment.

All values are reported as least square means. When there were significant (*p* < 0.05) effects or tendencies (*p* ≤ 0.10), pairwise comparisons were performed with Tukey–Kramer adjustments.

## 3. Results

Latency to use any brush after entering the pen was 4.0 ± 8.4 min (mean ± SD), with individual latency ranging from 0.1 to 31.1 min and 0.1 to 58.4 min in the 2- and 4-brush treatments, respectively. In the 4-brush treatment, brushes located near the feeder were used first 75% of the time. 

All brush-use behaviors will be discussed based on main effects related to brush treatment and period, as there were no significant interactions (*p* ≥ 0.17). 

### 3.1. Brush Quantity

**Figure 4:**
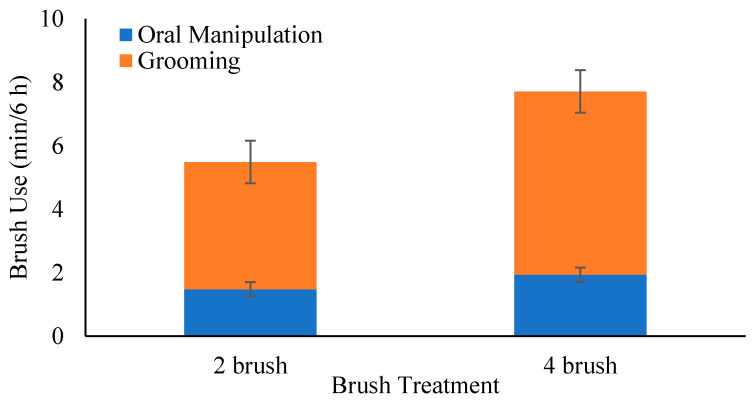
The mean ± SE duration of brush use for oral manipulation and grooming, averaged per 6 h period of observation, for each brush treatment (2 vs. 4 brushes provided to 7 to 8 group-housed heifers). The error bars represent the standard error of the means.

The number of brushes provided to group-housed heifers did not impact the duration of oral manipulation, grooming, or total brush use (*p* ≥ 0.10; Figure 4). Heifers provided with more brushes exhibited longer bouts (*p* = 0.029), but there was no treatment difference observed for bout frequency (Table 1).

### 3.2. Brush Use across Time of Exposure

**Figure 5:**
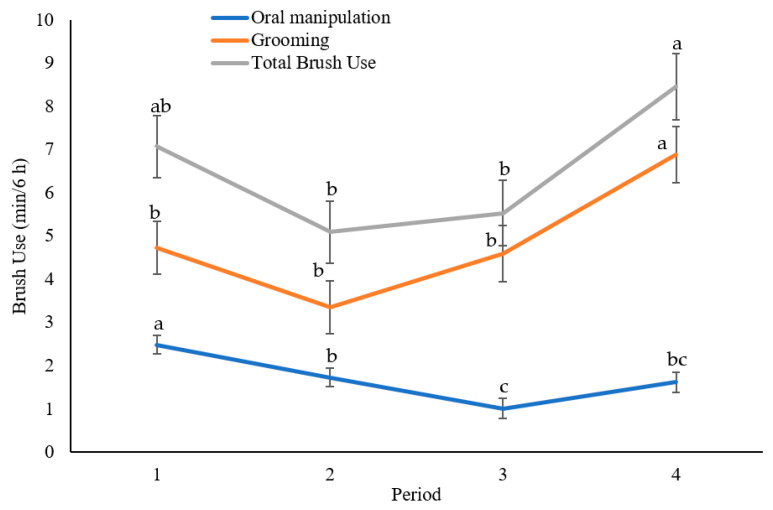
The mean ± SE duration of oral manipulation, grooming, and total brush use (the sum of the two aforementioned subsets) for each observed period (periods 1–4: 0–6, 18–24, 120–126, and 138–144 h after exposure, respectively), regardless of brush treatment. Letters within the same-colored line indicate significant (*p* < 0.05) pairwise differences between periods.

Total brush use in the final observation period (138–144 h of exposure) was greater than after 18–24 and 120–126 h of exposure (*p* ≤ 0.007), but it was similar to the first observation period (0–6 h of exposure, *p* = 0.28; Figure 5). Heifers tended to exhibit greater total brush use in the initial 6 h compared to 18–24 h of exposure (*p* = 0.052), but it remained similar during 120–126 h of exposure (*p* = 0.20). The greater duration of total brush use in the initial 6 h was facilitated by greater durations of oral manipulation compared to all other periods (*p* ≤ 0.027; Figure 5). Oral manipulation decreased from 18–24 h to 120–126 h of exposure (*p* = 0.045), but then remained consistent in 138–144 h compared with the previous two periods (*p* ≥ 0.12). Despite the decrease in oral manipulation from the initial to the final 6 h period, the consistent levels of total brush use between those periods were facilitated by greater durations of grooming in the final observation period (138–144 h of exposure) compared to all other periods (*p* ≤ 0.026; Figure 5). 

Bouts were more frequent in the initial 6 h of exposure than during 120–126 h of exposure (*p* = 0.007; Table 2). Bout frequency was similar among the other periods (*p* ≥ 0.21), although bouts tended to be more frequent during 18–24 h than in 120–126 h of exposure (*p* = 0.051). Bout duration was similar within the novel (0–6 and 18–24 h of exposure) and non-novel periods (120–126 and 138–144 h of exposure), respectively (*p* ≥ 0.88); overall, heifers exhibited shorter bouts during the novel compared to the non-novel period (*p* < 0.001; Table 2).

### 3.3. Competition

**Figure 6:**
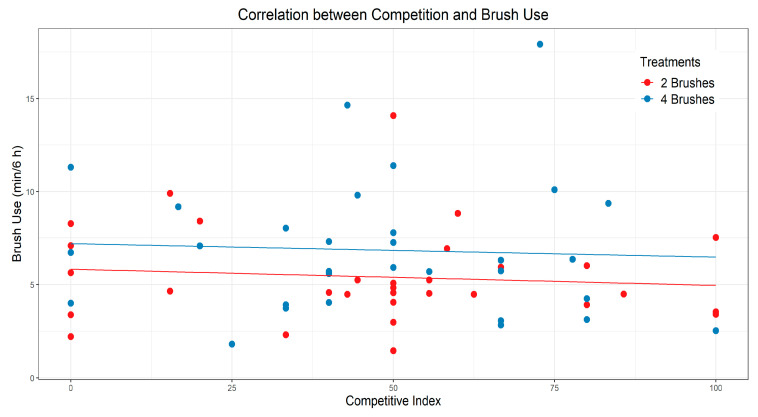
Correlation between individual heifer competitive index value (calculated as frequency of initiated displacements (actor role) divided by the total number of displacements she was involved in (actor and receiver roles), multiplied by 100) and the duration of brush use (average per 6 h period). One heifer in the 4-brush treatment was uninvolved in displacements and was excluded. Pearson correlations were performed regardless of treatment (R = −0.07, *p* = 0.61) and within each treatment (2-brush: R = −0.10, *p* = 0.58; 4-brush: R = −0.05, *p* = 0.78).

The quantity of brushes had no impact on the number of displacements (*p* = 0.75, Table 1). Fewer displacements occurred during 120–126 h of exposure compared to the initial 6 h after exposure (*p* = 0.003; Table 2) and 138–144 h of exposure (*p* = 0.039), but it remained similar during 18–24 h of exposure (*p* = 0.55). No correlation was observed between individual competitive index values and brush use within or across treatments (*p* ≥ 0.58; Figure 6).

## 4. Discussion

### 4.1. Overall Brush Use

Upon first exposure, naïve heifers began using brushes nearly immediately, with average latency similar to the previously reported results for group-housed heifers naïve to stationary brushes (3.4 ± 4.9 min, ranging from 0.1 to 17.8 min; [13]), but individual maximums were greater in the current study. This difference could indicate that certain heifers may not have had enough voluntary access in the current study, with one brush provided for every two or four heifers, compared to one brush per heifer in Van Os et al. [13]. However, the greatest latency in our study was observed in the four-brush treatment, which could alternatively reflect variation in individual heifer motivation to access the brush upon entrance into the novel pen. In addition to individual variation in latency, heifers in the current study showed variation in which brush they first contacted in the four-brush treatment, using brushes closest to the feeder 75% of the time. This initial use of brushes near the feed bunk was consistent with previous work [13], although those authors reported that initial preference did not continue after the first day of observation.

Providing two vs. four brushes for a group of eight heifers did not impact the duration of brush-directed behaviors (oral manipulation, grooming, or total brush use). Heifers used the brushes for grooming over 73% of the time, with the remainder spent orally manipulating the brushes. This distribution is similar to other studies that reported cattle used brushes for grooming for the majority of the observation time (>60% in weaned heifers [13]; >70% in pre-weaned calves [10]). This consistency among studies suggests that stationary brushes provide an appropriate outlet for both of these behaviors, but that grooming potentially has a higher motivation when using brushes.

The current study is the first to evaluate the effects of different brush quantities on brush use in any age of cattle. Van Os et al. [13] observed brush use in two different scenarios with four brushes per four heifers and four brushes per two heifers; however, no direct evaluation of stocking density was performed. The previous study reported, on average, greater total stationary brush use when four brushes were provided to four heifers compared to our findings using a 1:4 ratio, while remaining similar to our findings using a 1:2 ratio. On the contrary, lesser total brush use was reported when four brushes were provided to two heifers [13], although this was most likely due to limited access with the brushes located inside the freestalls. Studies involving only one brush provided to a group [11] or a 1:1 ratio for individually housed dairy calves [10,30] reported, on average, lesser total stationary brush use compared to the levels observed in our study, which may relate to the age of the animals or other differences in housing management. One exception was a study in which greater brush use was observed when nine beef steers were provided one large stationary brush structure [21]; however, this may be explained by the massive size of the structure, with 12 brushes combined, compared to only a single stationary brush in the other studies.

Overall, total brush use was greater in the first and final observation periods (0–6 and 138–144 h of exposure). This pattern was driven by the greatest duration of oral manipulation in the first period and the greatest duration of grooming in the last period. We speculate that the greater oral manipulation after initial brush exposure may have reflected heifers’ exploration of their new environment. In previous studies, group-housed heifers [13] and beef steers [21] used stationary brushes the most within the first 24 h, after which brush use declined and stabilized. In the former study, oral manipulation was stable across 6 d, whereas grooming decreased after the first day of observation [13], which the authors speculated reflected heifers’ initially greater urge to groom themselves due to the lack of appropriate scratching surfaces in their previous housing environment. Our findings of greater grooming in the final observation period may have reflected learning and increased motivation to perform this natural behavior after heifers recognized the brushes as an appropriate outlet for this type of behavior. Regardless of the temporal variation we observed across periods, all heifers continued to use brushes through the final observation period. Likewise, continued use of stationary brushes has been documented in group-housed heifers observed across 13 d [13] and in beef steers for 22 and 64 d [21,24]. These patterns among studies suggest that cattle remain motivated to use brushes after they are no longer novel. This supports the importance of providing stationary brushes as appropriate, beneficial outlets for grooming and other behaviors to promote animal welfare. 

### 4.2. Bout Characteristics

Patterns of brush use by cattle involve short durations separated by short breaks. Defining a bout criterion improves biological interpretation of brush-use events. Horvath and Miller-Cushon [9] performed the first calculation of brush bout criteria in cattle (group-housed, pre-weaned calves with a rotating brush), and those authors later applied the method to individually housed pre-weaned calves with stationary brushes [10] and group-housed beef steers with a mechanical brush [23]. In our study, we used this methodology to calculate criteria for each six-hour period (ranging from 20.0 to 125.9 s), which we pooled into two periods when brushes were more vs. less novel (25.1 vs. 63.1 s, respectively), based on descriptive similarity in their criteria. In previous studies in which bout criteria were calculated, inter-bout criteria were longer (125.9 s in pre-weaned calves using a rotating brush [9]; 158.5 and 251.1 s in two experiments with weaned beef steers using a mechanical brush [23]; 13.2 min in individually housed calves using a stationary brush [10]). These data do not suggest a clear pattern between inter-bout criteria and the ratio of cattle to brushes, but rather suggest that bout characteristics may vary depending on other management or individual animal variation. The variation across studies may also be associated with behavioral observation methods, such as when only select focal individuals are observed in a group setting or when time sampling is used instead of continuous observation [23].

Our bout analysis revealed brush use frequencies between 12.2 and 18.8 bouts per 6 h, which lasted 19.9 to 41.6 s per bout. Patterns of brush use previously reported in the literature vary, in part because some studies described visits (raw counts of brush use events) as opposed to bouts defined with a bout criterion. For example, pair-housed dairy calves with one mechanical brush displayed 94 events per 20 h, each event lasting 17.8 s [8]. In contrast, when studies have explicitly calculated bout characteristics, fewer but longer bouts of brush use were reported in group-housed dairy calves (10.4 bouts/12 h lasting 154.2 s/bout [9]), beef steers (7 to 8 bouts/d lasting 4 to 6 min/bout in 2 experiments [23]), and individually housed dairy calves (7 to 8 bouts/12 h lasting 6 to 8 min/bout [10]). In addition to selecting appropriate behavioral observation methods for capturing brief brush-use events, calculating bout characteristics should be considered for future studies of brush use to most accurately report and interpret findings.

Within our study, access to more brushes (1:2 vs. 1:4 brush-to-heifer ratio) resulted in longer bouts (35.1 vs. 27.5 s/bout, respectively), which could reflect more opportunity for simultaneous use, and thus expression of more complete bouts. This could imply that, to promote animal welfare by providing the best opportunities for heifers to perform more complete bouts of brush use, farmers should consider providing a high ratio of brushes to groups when possible (e.g., one brush per 1–2 heifers). However, across other studies, brush stocking density does not appear to explain bout length. In the absence of competition, individually housed dairy calves exhibited much longer bouts (6 to 8 min/bout [10]) than in our study. However, this was also the case in other studies with only one brush per group of four dairy calves (154.2 s/bout [9]) and four or eight beef steers (4.2 and 5.6 min/bout, respectively [23]). The longer bouts in past studies compared to ours may be related to environmental factors, such as pen size and brush type or location relative to other resources, rather than just group size. In our study, two brushes in each treatment were located near the feed bunk, and an additional brush in the four-brush treatment was near the waterer, which may have facilitated short bouts of brush use while heifers used nearby resources. Lactating cows have been shown to be similarly motivated to access a brush and fresh feed [1]. Perhaps because heifers in our study could all occupy the feed bunk at once, they had more opportunity to switch back and forth between resources compared to in a more competitive feeding environment; this hypothesis could be evaluated in future studies. Finally, the heifers in our study were provided unchopped hay, which may have affected bout length due to the interconnectedness of oral behaviors; Horvath et al. [10] observed that individually housed dairy calves fed chopped bermudagrass had shorter bouts of brush use than those without hay. 

In addition, we evaluated bout characteristics over time because we predicted that bout frequency and duration would change as the novelty of the brushes decreased. Overall, we found that bouts were shorter and more frequent upon initial brush exposure and lengthened and became less frequent as novelty decreased after the first day. These patterns could reflect the transition from short, initial oral exploratory behavior to longer, sustained use of the brushes for grooming. The temporal patterns may also suggest that the provision of multiple brushes provided opportunity for voluntary brush use without constant interruption and competition for use. In a previous study, group-housed pre-weaned dairy calves similarly performed more bouts during week four of life compared to later weeks, although bout length did not change [9]. Ours is the first study to evaluate changes in bout characteristics beginning with the first exposure to brushes, and further research is needed to understand how and why bout characteristics change after initial use. 

### 4.3. Competition

The quantity of brushes had no impact on the number of competition events. Overall, displacements were relatively infrequent, occurring at an average rate of one per hour. In previous work by our group on weaned heifers with access to one brush per heifer (four heifers per bedded-pack pen), fewer than four displacements occurred in the first 24 h of exposure, decreasing to two displacements per day on subsequent observation days [13]. During the second phase of the same study, in which two brushes were available per heifer (two heifers per freestall pen), only one displacement event was observed across two days of observation [13]. In contrast, many other studies provided only one brush per larger group of cattle (e.g., refs. [9,11,12,14]), but to date, only three have reported competitive behavior. For groups of lactating cows with one mechanical brush, less than one displacement per two hours was reported for groups of 12 [2,22], and less than one displacement per hour occurred in groups of 20 [16]. Although the rate of displacements reported in the literature has been relatively low, patterns among studies suggest a possible relationship with the ratio of cattle to brushes. More research is needed on the effects of brush availability on competition and brush use when the ratio of cattle to brushes is greater than in our studies on small groups of heifers, particularly for stationary brushes, which may be more economically feasible for farms to provide in quantities greater than one per group of cattle. Furthermore, displacements are one of many possible measures of competition, which may also involve non-physical interactions, such as subordinate individuals rescheduling use to avoid contact with dominant individuals.

Contrary to our prediction, the calculated competitive index for each heifer showed no correlation with individual brush use. This could be because heifers who displaced another conspecific may not have intended to use the brush, and the contact could have been accidental, especially at brushes located near other resources such as the feed bunk and waterer. Nonetheless, intent cannot be assumed during behavioral observation, and all physical contacts between heifers resulting in cessation of brush use were coded as displacements. In previous work on lactating cows, a low correlation was likewise reported between a competitive success index and brush use [22]. Therefore, a competitive index may not always reflect motivation to use a brush. Another approach for characterizing social dynamics is to classify cattle as dominant or subordinate. Foris et al. [16] did so for lactating cows based on successful replacements at the feed bunk and water troughs. Although the number of displacements from a mechanical brush did not vary between dominant or subordinate cows, the former used the mechanical brush more during peak feeding time and overall [16]. This suggests that dominance plays a role in brush access and supports the idea that subordinate individuals may choose to avoid physical competition for the resource. 

## 5. Conclusions

Our study is the first to evaluate the role of stocking density on brush use and competition in any age class of cattle. Brush quantity did not impact the overall duration of brush use or competition. However, heifers provided with more brushes performed longer bouts, suggesting that the provision of four brushes to a group of eight heifers provided greater opportunity for uninterrupted brush use. Naïve heifers used brushes soon after exposure, with the greatest oral manipulation of brushes in the initial 6 h, perhaps reflecting exploration. In contrast, grooming, which comprised the majority of brush use, was greatest at the end of the sixth day after brush exposure. The continued use of brushes over time by all heifers illustrates the importance of providing appropriate outlets for both of these important natural behaviors to promote animal welfare.

## Figures and Tables

**Table 1:** Brush-directed behaviors performed by group-housed heifers (7 to 8 heifers/group) for each treatment (2 vs. 4 brushes/pen, *n* = 4 groups/treatment), averaged among four 6 h observation periods unless otherwise indicated.

Variable	Treatment		SE ^1^	*F*-Value ^2^	*p*-Value
	2 Brush	4 Brush			
Competition events (no.)	5.7	6.2	1.02	0.8 (1,6)	0.75
Competitive index ^3^	47.5	48.5	2.07	0.1 (1,6)	0.75
Bout Frequency (no.) ^4^	14.6	17.4	1.79	1.2 (1,6)	0.31
Bout Duration (s/bout) ^4^	27.5	35.1	1.85	8.5 (1,5.7)	0.029

^1^ Pooled standard error. ^2^ Degrees of freedom associated with each F statistic are reported in parentheses. ^3^ Frequency of initiated displacements (actor role) divided by the total number of displacements the individual heifer was involved in (actor and receiver roles), multiplied by 100; calculated on an individual heifer basis across the entire study. ^4^ Bout characteristics based on 2 pooled inter-bout criteria calculated from 0–6 and 18–24 h of exposure (periods 1 and 2, when the brushes were most novel) vs. 120–126 and 138–144 h of exposure (periods 3 and 4).

**Table 2:** Brush-directed behaviors performed by group-housed heifers (7 to 8 heifers/group) for each 6 h observation period, regardless of brush quantity treatment (2 vs. 4 brushes/pen, *n* = 4 groups/treatment).

	Period ^1^						
Behavior	1	2	3	4	SE ^2^	*F*-Value ^3^	*p*-Value
Competition events (no.)	8.6 ^a^	4.9 ^bc^	3.1 ^b^	7.1 ^ac^	1.08	6.9 (3,16.4)	0.003
Bout Frequency (no.) ^4^	18.8 ^a^	17.1 ^ab^	12.2 ^b^	15.8 ^ab^	14.50	5.2 (1,16.3)	0.011
Bout Duration (s/bout) ^4^	22.5 ^b^	19.9 ^b^	41.2 ^a^	41.6 ^a^	2.60	20.2 (1,16.7)	<0.001

^1^ Behavior observations were performed across four 6 h periods, defined as periods 1–4 (0–6, 18–24, 120–126, and 138–144 h after exposure, respectively). ^2^ Pooled standard error. ^3^ Degrees of freedom associated with each F statistic are reported in parentheses. ^4^ Bout characteristics based on 2 pooled inter-bout criteria calculated from 0–6 and 18–24 h of exposure (periods 1 and 2, when the brushes were most novel) vs. 120–126 and 138–144 h of exposure (periods 3 and 4). ^a–c^ Superscripts that differ within row indicate significant differences (*p* < 0.05).

## Data Availability

None of the data were deposited in an official repository, but information can be available upon request.
